# Joint embedding of biological networks for cross-species functional alignment

**DOI:** 10.1093/bioinformatics/btad529

**Published:** 2023-08-26

**Authors:** Lechuan Li, Ruth Dannenfelser, Yu Zhu, Nathaniel Hejduk, Santiago Segarra, Vicky Yao

**Affiliations:** Department of Computer Science, Rice University, Houston, TX 77005, United States; Department of Computer Science, Rice University, Houston, TX 77005, United States; Department of Electrical and Computer Engineering, Rice University, Houston, TX 77005, United States; Department of Computer Science, Rice University, Houston, TX 77005, United States; Department of Electrical and Computer Engineering, Rice University, Houston, TX 77005, United States; Department of Computer Science, Rice University, Houston, TX 77005, United States

## Abstract

**Motivation:**

Model organisms are widely used to better understand the molecular causes of human disease. While sequence similarity greatly aids this cross-species transfer, sequence similarity does not imply functional similarity, and thus, several current approaches incorporate protein–protein interactions to help map findings between species. Existing transfer methods either formulate the alignment problem as a matching problem which pits network features against known orthology, or more recently, as a joint embedding problem.

**Results:**

We propose a novel state-of-the-art joint embedding solution: Embeddings to Network Alignment (ETNA). ETNA generates individual network embeddings based on network topological structure and then uses a Natural Language Processing-inspired cross-training approach to align the two embeddings using sequence-based orthologs. The final embedding preserves both within and between species gene functional relationships, and we demonstrate that it captures both pairwise and group functional relevance. In addition, ETNA’s embeddings can be used to transfer genetic interactions across species and identify phenotypic alignments, laying the groundwork for potential opportunities for drug repurposing and translational studies.

**Availability and implementation:**

https://github.com/ylaboratory/ETNA

## 1 Introduction

Many critical discoveries in medicine have been uncovered by molecular studies conducted in model organisms, and the importance of leveraging these models for translational studies only continues to increase (Aitman *et al.* 2011). However, one of the major challenges to realizing the full potential of model organism studies is functional knowledge transfer (Park *et al.* 2013), the process of translating information learned in one species to another.

Model organisms are an important, well-established tool for studying fundamental biological pathways and disease etiology, especially given the technical and ethical limitations of performing direct research on humans (O’Neil *et al.* 2017). Furthermore, their inherent characteristics and range of available assays provide opportunities to capture unique biological perspectives that would otherwise be impossible. For example, synthetic lethality studies in yeast (Tong *et al.* 2004) reveal genetic interaction relationships, genetic screens in organisms with short life cycles such as worm and fly (Brenner 1974, St Johnston 2002) can be used to study aging-related phenotypes that are much costlier to study in other organisms, and sophisticated optogenetics and behavioral assays can be used to study complex neurobiology in mice (Fenno *et al.* 2011). The ability to transfer meaningful molecular insights from one species to another is therefore a central problem encountered by many experimental biologists, critical for both helping reveal the broader implications of individual studies as well as guiding the generation of new hypotheses for further experimentation.

One intuitive approach to transfer findings from one organism to another is via sequence similarity, attributing the same biological function to orthologous genes. However, across organisms, similar pathway-specific functions can be taken on by proteins that may not be the most sequence similar (Park *et al.* 2013). For this reason, simply assigning conserved function between species through orthologous proteins is insufficient. To address this problem, researchers have developed network alignment methods to consider similarity across protein–protein interaction (PPI) networks from different organisms or combine orthology together with PPI networks, to better capture conserved protein function. Intuitively, if two proteins maintain similar interaction partners, they are more likely to play similar functional roles in their respective organisms. Several PPI network alignment methods have been developed (Table 1), including methods that leverage PageRank (Singh *et al.* 2008, Kalecky and Cho 2018), genetic algorithms (Vijayan *et al.* 2015), or search algorithms (Patro and Kingsford 2012, Neyshabur *et al.* 2013, Mamano and Hayes 2017), as well as hub-alignment (Hashemifar and Xu 2014) and graphlet-based methods (Malod-Dognin and Pržulj 2015). However, of the existing alignment methods that use both sequence similarity and PPI networks, the vast majority usually optimize a convex combination of the two.

**Table 1.** Comparison of ETNA with existing unsupervised pairwise network alignment methods.

Method	Interpretable embeddings	Cross-species anchors	Combining sequence and topology	Directionality	Algorithm	Code availability
ETNA	Yes	Orthologs	Nonlinearly	Bidirectional	Autoencoders	Python
MUNK (Fan *et al*. 2019)	Yes	Orthologs	Nonlinearly	Directional	SVD	Python
IsoRank (Singh *et al*. 2008)	No	BLAST	Linearly	Bidirectional	PageRank	Linux executable
HubAlign (Hashemifar and Xu 2014)	No	BLAST	Linearly	Bidirectional^a^	Minimum-degree heuristic algorithm	C++
PrimAlign (Kalecky and Cho 2018)	No	BLAST	Linearly	Bidirectional	Markov chain + PageRank	Not available
SANA (Mamano and Hayes 2017)	No	BLAST	Linearly	Bidirectional^a^	Simulated annealing	Webserver, C++
L-GRAAL (Malod-Dognin and Pržulj 2015)	No	BLAST	Linearly	Bidirectional	Lagrangian graphlet	C++, not working
GHOST (Patro and Kingsford 2012)	No	GO	Does not use sequence	Bidirectional^a^	Seed-and-extend with local search	C++
NETAL (Neyshabur *et al*. 2013)	No	GO	Does not use sequence	Bidirectional^a^	Greedy search on similarity matrix	Webserver
MAGNA++ (Vijayan *et al*. 2015)	No	n/a	Does not use sequence	Bidirectional	Genetic algorithm	Executable, C++

a The method has a bidirectional objective function, but the returned output is directional.

The main limitation of formulating the alignment problem in this manner is that network topology and sequence similarity are, in some sense, pitted against each other (Fan *et al.* 2019, Gu and Milenković 2020). A recent method, MUNK, seeks to address this challenge by reframing the alignment problem as a joint embedding problem (Fan *et al.* 2019). Specifically, it uses a regularized Laplacian kernel to embed each PPI network individually and uses orthology to project one embedding to the other, achieving better performance than traditional biological network alignment methods for functional tasks. However, MUNK’s embeddings are directional, requiring a decision between “source” and “target” species, leading to differing results for the alignment between the same pair of species. Furthermore, MUNK simply uses the same dimensionality as the smaller of the two PPI networks for its latent embedding, which does not fully take advantage of the natural “compression” that is inherent to embedding methods and makes it prone to overfitting.

**Figure 1.**
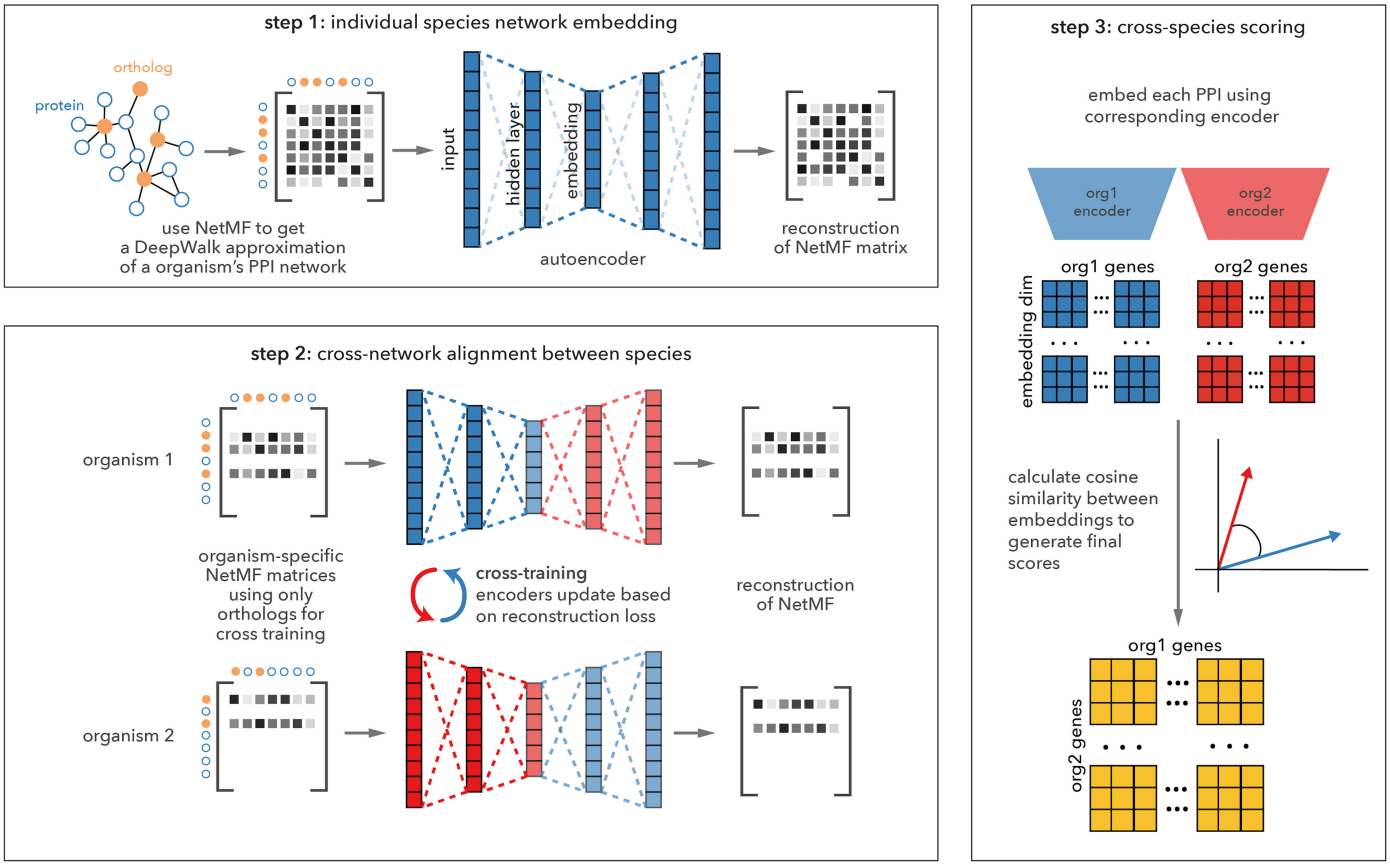
Overview of the ETNA method. ETNA’s framework is roughly divided into three main parts: (1) training an autoencoder to embed PPI networks, (2) aligning the embeddings between species using orthologous proteins as anchors for cross-training, and (3) scoring pairs of genes across organisms based on their cosine similarities in embedding space. The embeddings and cosine similarities can both be used for downstream tasks to predict functional similarity across species.

Here, we present Embedding to Network Alignment (ETNA), a deep learning method for estimating functional relevance between genes from different species (Fig. 1). ETNA presents a novel autoencoder architecture to create individual network embeddings that preserve the global and local topological structures of biological networks. Taking inspiration from advances in natural language processing (NLP), ETNA swaps the encoders and decoders of two embeddings in a cross-training framework (Lauly *et al.* 2014) while using orthologous genes as anchors to align the embeddings to a joint latent space. The alignment process also allows information from the other species to refine each individual network embedding. ETNA’s final output is a bidirectional joint embedding that encodes both within and between species functional relationships.

Using human and four other commonly studied model organisms (mouse, yeast, fly, and worm), we show that ETNA’s joint embedding can capture both pairwise and group functional relationships across species significantly better than existing methods. We further explore applications of the joint embedding, including predicting genetic interactions, identifying potential phenotypic alignments between human and mouse, as well as providing new insights on relationships between human disease, mouse phenotypes, and drug targets.

## 2 Materials and methods

### 2.1 A framework for cross-species network alignment

To align two networks, ETNA iterates between two main steps: (1) calculating an embedding for each individual network; and (2) aligning the two embedding spaces using orthologs as reference anchors (steps 1 and 2 in Fig. 1). By doing so, ETNA identifies a joint embedding between two networks. When applied to PPI networks, this joint embedding can be interpreted as a latent functional similarity map and enables reasoning about relationships between proteins across organisms, beyond ortholog pairs.

#### 2.1.1 Individual network embeddings

ETNA uses an autoencoder framework to generate lower-dimensional latent embeddings that preserve both local and global network topology while capturing the nonlinear relationships in the input network. As in many other real-world networks, gene relationships are diverse and complex, with many nonlinear interactions (Kitano 2002). The capacity for autoencoders to capture complex nonlinear relationships through their activation functions renders them a well-suited model for gene functional relationships. The framework is agnostic to the type of network, but here we focus on PPI networks. Specifically, given a PPI network, we represent it as an undirected graph G=(V,E), where $V=\{v_{1},v_{2},\ldots ,v_{n}\}$ is the set of *n* proteins and *E* is the set of reported physical interactions between pairs of proteins.

Importantly, instead of using *G*’s adjacency matrix *A* directly as input to the autoencoder, ETNA uses a closed-form approximation of the random walk process on *G*, which we denote by *M* (Equation 3). This matrix can consider long-range topology that an adjacency matrix ignores. The autoencoder is composed of two parts, namely an encoder and a decoder. For each vertex $v_{i}$, the encoder compresses the *n*-dimensional input (i.e. the *i*th row of *M*, denoted by $m_{i}$ in the following equations) through a 1024-dimensional hidden layer to a 128-dimensional latent embedding $z_{i}$. We collect vectors $z_{i}$ for different vertices into a matrix $Z\in R^{n\times 128}$ as its rows. The decoder then uses an independent 1024-dimensional hidden layer to map the latent embedding back into reconstruction space (step 1 in Fig. 1). To capture local and global network structure, ETNA uses the following objective function:


(1) $$L_{\text{embed}}=\alpha L_{\text{high}}+\gamma L_{1\text{st}}+\lambda L_{2}+\omega L_{\text{norm}}.$$

The $L_{1\text{st}}$ loss preserves the local first-order structure of the PPI network by maximizing the similarity of embeddings between vertices that are directly connected. On the other hand, $L_{\text{high}}$ captures global network topology by modifying the standard autoencoder reconstruction loss to encourage proteins that have similar network relationships to have similar embeddings. These two losses are described in more detail below. We also consider two regularization terms: $L_{2}$ norm on the autoencoder parameters to avoid overfitting and $L_{\text{norm}}=\sum_{i=1}^{n}\|z_{i}{\|}_{2}^{2}$ to avoid exploding norms. All hyperparameters are tuned by cross validation (Section 2.1.4).

#### 2.1.2 Preserving local first-order structure

In most real-world networks, the presence of a shared edge between two vertices is a strong signal of similarity (Tang *et al.* 2015), and this is true in PPI networks as well. Two proteins that physically interact with each other are more likely to be performing similar functions. The *first-order proximity* captures this local pairwise structure between two vertices. In ETNA, for a pair of vertices $\left(v_{i},v_{j}\right)$ connected by an edge, their latent embeddings should be similar. Thus, the objective function for *first-order proximity* is defined as:


(2) $$L_{1\text{st}}=\frac{1}{2|E|}\sum_{i=1}^{n}\sum_{j=1}^{n}A_{i,j}\cdot \;\text{log}(\text{cos}(z_{i},z_{j}))$$

where |E| is the number of edges in the graph, *n* is the number of vertices in the graph and $\text{cos}(z_{i},z_{j})=z_{i}z_{j}^{⊤}/(\|z_{i}{\|}_{2}\cdot \|z_{j}{\|}_{2})$ reflects the similarity between $v_{i}$ and $v_{j}$ in the latent embedding space. This objective function can be considered as half of the traditional cross entropy formulation and only considers the positives (i.e., reported PPIs). There are two important reasons for this: (i) Physical interactions are not the only criteria to determine whether two proteins are functionally similar, and (ii) PPI network data are not yet complete and definitely have false negatives (Supplementary Table S2). Therefore, ETNA focuses on capturing the presence of reported PPIs and minimizes the contribution of missing PPIs.

#### 2.1.3 Preserving global structure through higher-order proximity

Given a network, one straightforward way of calculating an embedding using autoencoders is using the corresponding row of the adjacency matrix as input for an individual vertex (Wang *et al.* 2016), essentially embedding the network neighborhood of each vertex. However, this formulation only considers direct and 2-hop neighbors (i.e., “friends of friends”) and ignores longer-range network topology. This is potentially problematic as there are well-studied biological relationships, such as the MAPK pathway (Seger and Krebs 1995), that can easily involve more than a chain of three genes. In the past decade, random walk-based methods such as DeepWalk (Perozzi *et al.* 2014) have been shown to learn latent representations that successfully capture network topology. Recently, the NetMF method has been proposed based on a closed-form estimate of the similarity matrix that is implicitly factorized in DeepWalk (Qiu *et al.* 2018). Compared to the adjacency matrix, the NetMF matrix not only contains information for direct connections, but also contains similarity values between vertices that are not directly connected. Using its rows as input enables ETNA to consider the higher-order proximity of the PPI network. The NetMF matrix $M\in R^{n\times n}$ is calculated from the adjacency matrix *A* as in (Qiu *et al.* 2018):


(3) $$M=\text{log}\;\left(\frac{\text{vol}(G)}{T}\left(\sum_{i=1}^{T}{\left(D^{-1}A\right)}^{i}\right)D^{-1}\right)-\text{log}\;b,$$

where *D* is the diagonal degree matrix whose entry $D_{i,i}$ is $v_{i}$’s degree, $\text{vol}(G)=\sum_{i=1}^{n}D_{i,i}$ is the volume of graph *G*, *T* is the context window size, and *b* is the negative sampling parameter. Intuitively, $M_{i,j}$ can be considered as a weighted count of the number of paths from $v_{i}$ to $v_{j}$ that have lengths no >*T*. ETNA uses the rows of *M* as input into its autoencoder and estimates a reconstruction $\hat{M}$ from the latent embedding. Good reconstructions imply that *higher-order proximity* has been well-captured in the latent embedding, and thus the objective function is defined as:


(4) $$L_{\text{high}}=\text{BCE}(\sigma (\hat{M}),M),$$

where BCE is the binary cross entropy, defined as $\mathrm{BCE}(Y^{\prime },Y)=\frac{1}{n}\sum_{i=1}^{n}(Y_{i}\cdot \;\text{log}\;Y_{i}^{\prime }+(1-Y_{i})\cdot \;\text{log}(1-Y_{i}^{\prime }))$, and σ is the sigmoid activation function. The use of the sigmoid here is akin to having a sigmoid activation function prior to estimating the reconstruction error, but incorporating it directly into the loss estimation improves numerical stability.

#### 2.1.4 Neural network architecture

A fixed architecture is used for the autoencoders (1 hidden layer, with an embedding dimension of 128), and the same activation function (LeakyReLU with a negative slope of 0.1) is used throughout. A simple exploration of different neural network architectures demonstrates that the number of hidden layers and embedding dimensions did not affect prediction performance much, especially considering computational tradeoffs. While ReLU offers similar performance as LeakyReLU, other choices of activation function result in worse performance (Supplementary Fig. S1).

#### 2.1.5 Cross-species network alignment using orthologs

To align the two previously independent embeddings, we use orthologous proteins as “anchors” between two different species. The underlying intuition is to encourage orthologous protein pairs from the two species to have similar latent features, all while keeping the distance relationship between vertices within each network. Instead of arbitrarily assigning source and target networks and having a directional projection, we use a cross-training method that pushes the embeddings of both networks toward a joint latent space simultaneously (step 2 in Fig. 1). The final embedding in the joint latent space thus contains distance relationships between proteins across networks.

Specifically, the alignment process uses a cross-training method inspired from language translation methods developed in NLP (Lauly *et al.* 2014). Recall that we defined a PPI network as an undirected graph G=(V,E), where |V|=n. Now, given a second PPI network $G^{\prime }=(V^{\prime },E^{\prime })$ with $|V^{\prime }|=n^{\prime }$ and a set of orthologous pairs $\Theta \subset V\times V^{\prime }$, consider an orthologous pair $(\theta_{i},\theta_{i^{\prime }})\in \Theta$: if $\theta_{i}$ and $\theta_{i^{\prime }}$ play similar roles in their respective networks (i.e., the same “word” in different languages), then we seek to identify a joint embedding (i.e., latent semantic space in NLP or latent functional space for PPIs). If such a joint embedding exists, then one way of thinking about the “translation” task is that once *G*’s encoder places $\theta_{i}$ in the joint embedding, then $G^{\prime }$’s decoder should be able to reconstruct $\theta_{i^{\prime }}$’s neighborhood structure, and vice versa. This intuition leads to the following objective function:


(5) $$\begin{array}{c} L_{\text{cross}}=\phi *\sum_{i,i^{\prime }|(\theta_{i},\theta_{i^{\prime }})\in \Theta }\text{BCE}(\sigma ({\text{De}}_{G^{\prime }}({\text{En}}_{G}(m_{i}))),{m^{\prime }}_{i^{\prime }})+\\ \text{BCE}(\sigma ({\text{De}}_{G}({\text{En}}_{G^{\prime }}({m^{\prime }}_{i^{\prime }}))),m_{i}), \end{array}$$

where $\left\{m_{1},\ldots ,m_{n}\right\}$ and $\left\{m_{1}^{\prime },\ldots ,m_{n^{\prime }}^{\prime }\right\}$ are the row vectors of the corresponding NetMF matrices of *G* and $G^{\prime }$, EN and DE are abbreviations for encoder and decoder respectively.

The alignment process only updates the weights in the encoders, since the alignment should happen in the latent space rather than the reconstruction space. The training process iterates between the embedding and alignment steps to generate a joint embedding that preserves both within- and between-species closeness. The reasoning here is that well-trained individual network embeddings are too “rigid” to accommodate between-species protein closeness. Therefore, one of embedding process followed by one epoch of alignment process is considered as one training block for ETNA, and the number of training block epochs is determined by cross-validation (Section 2.1.4).

#### 2.1.6 Calculating the cross-species score matrix

After all training epochs are complete, the PPI embeddings generated for each individual species are in a joint latent space and can thus be compared directly. Here, we use the cosine similarity between each pair of proteins across the two species as a similarity score. More precisely, we compute a score matrix $S=\text{cos}(Z,Z^{\prime })\in R^{n\times n^{\prime }}$, where $\text{cos}(A,B)=\frac{A\cdot B}{\left||A||||B|\right|}$. The score matrix, *S*, contains pairwise similarities between all vertices in *G* and $G^{\prime }$ (step 3 in Fig. 1).

#### 2.1.7 Hyperparameter tuning

For a given joint embedding, there are nine main hyperparameters in the loss functions that are tuned: α, γ, λ, ω for each of the two individual network embeddings described in Equation (1), and ϕ at the cross training stage described in Equation (5).

The search space for these nine hyperparameters is explored on a logarithmic scale (base 10). α controls the contribution of $L_{\mathrm{high}}$, which preserves the entire neighborhood information of a vertex, so it is designed to be the dominant term in $L_{\mathrm{embed}}$. Therefore, α has the search space $\left[10^{0},10^{3}\right]$, whereas the search space for the auxiliary parameters is $\left[10^{-1},10^{2}\right]$.

To determine the set of optimal hyperparameters, Bayesian optimization using Gaussian Processes (as implemented in the scikit-optimize package) is used to search for the optimal set of hyperparameters from the search space with 100 calls under 5-fold cross validation. The performance of the ranked score matrix is optimized for using AUPRC, with the gold standard being the functional similarity labels generated by our GO slim, as described in Section 2.3. Importantly, though our GO-based gold standard consists of gene pairs, cross-validation folds were stratified by genes (including orthologous genes across species), and gene pairs where genes appear in separate folds were entirely excluded. This ensures that genes would only appear in individual folds and avoid data leakage across folds. The selected hyperparameters are noted in Supplementary Table S1.

During hyperparameter tuning, ETNA’s alignment performance on validation and test sets were very similar (Supplementary Table S4), suggesting that the tuning process is not resulting in overfitting. In general, ETNA is robust to the choice of hyperparameters and can consistently outperform existing methods even with random sets of hyperparameters (Supplementary Fig. S2).

By design, $L_{\mathrm{high}}$ is the dominant term in $L_{\mathrm{embed}}$, and as such, for a new pair of species, we suggest starting with the following hyperparameters: α=100, γ=5, ω=1, λ=1, and ϕ=50. The optimal number of epochs depends on the evolutionary distance between two species (# orthologs) and the available PPI information (network completeness). For species pairs with more input information: more complete PPI (e.g., *H. sapiens*–*S. cerevisiae*) and more ortholog pairs (e.g., *H. sapiens–M. musculus*), we propose to use # epochs = 10. For species pairs with less complete input information, we suggest a larger number of epoch = 20. Using these “default” parameters constitutes a good starting point and can achieve strong performance even without hyperparameter tuning (Supplementary Fig. S2).

### 2.2 PPI network and orthology data

PPI data for each of the six species studied in this work were downloaded from BioGRID (v3.5.187) (Stark *et al.* 2006). Using Entrez gene identifiers, we constructed an unweighted and undirected species-specific PPI network, filtering out self-loops. We also recursively filtered out vertices with the same neighborhood structure, since with only topological information as input, these are indistinguishable to our model. Because these vertices are indistinguishable (typically vertices that have a single edge), the filtered vertices are selected randomly.

Supplementary Table S2 provides several summary statistics for the PPI networks of each of the major model organisms. We see that the coverage and completeness for different organisms vary greatly. Unsurprisingly, the PPI network and functional information for *S. cerevisiae*, the organism most evolutionarily distant from human, is also the most complete across all species (evident in the % of the genome with at least one reported PPI, network density, as well as % of genes with at least one GO annotation). All other species have different trade-offs: a large proportion of the *H. sapiens* genome has been probed for PPIs, and despite its much larger genome size, it has the second most complete PPI network, but proportionally, it is also the species with the lowest % of genes with at least one GO annotation. The PPI networks of *M. musculus*, *D. melanogaster*, and *C. elegans* vary in coverage (with *D. melanogaster* having the largest proportion of genes having at least one reported PPI and *C. elegans* having the fewest), but all have similar levels of network density, reaffirming that in addition to the varying numbers of proteins that are missing from the PPI, even among the proteins with reported interactions, there are likely a large number of missing edges.

Orthology data were downloaded from OrthoMCL (v6.1) (Li *et al.* 2003), and they were mapped to Entrez gene identifiers. In addition to use orthologs as anchors for the cross-species network alignment, we also explored using gene pairs at various BLAST bit score cutoffs (Supplementary Fig. S1). The prediction performance is not drastically different, though, notably, overly conservative bit score cutoffs that severely limit the number of anchors does result in consistently poorer performance.

### 2.3 Building a cross-species functional evaluation standard

The Gene Ontology (GO) provides valuable gene function annotations across species, where genes annotated to the same terms can be considered as functionally similar. We used GO (16 July 2020) (Consortium 2004) as the gold standard to evaluate the ability of our embedding-alignment method to capture cross-species functional similarity. We restricted annotations to the Biological Process (BP) aspect, which describes the molecular activities of genes, and used low throughput experimental evidence codes (EXP, IDA, IMP, IGI, IEP), excluding evidence code IPI (Inferred from Physical Interaction) to avoid introducing any circularity to the evaluations. All GO annotations were propagated through the “is a” and “part of” relations. We restricted our set of GO terms to a slim set representing specific diverse functions that are present across *H. sapiens*, *M. musculus*, *S. cerevisiae*, *D. melanogaster*, and *C. elegans*. We defined specificity as terms with at least 10 genes and at most 100 genes annotated. This annotation-driven slim set was combined with expert-curated GO slim terms (Greene *et al.* 2015). Gene pairs from two different species were considered as positive labels if they shared at least one GO term in our selected slim set and negative otherwise.

We also wanted to evaluate our method on the full set of GO terms (without being restricted to a slim set). To do so, we calculated the Jaccard index between every pair of proteins, i.e., the fraction of GO terms annotated to both proteins in relation to the total number of GO terms annotated to either protein.

### 2.4 Predicting genetic interactions

Genetic interactions for *S. cerevisiae* and *S. pombe* were downloaded from BioGRID (v3.5.187) (Stark *et al.* 2006). Gene pairs with reported “Synthetic Lethality” were regarded as positive examples of Synthetic Lethality (SL). For each species, an equal number of negative examples were subsampled from pairs where both genes were present in the SL dataset but not reported to show a genetic interaction. Using this gold standard, we applied a support vector machine (SVM) (Cortes and Vapnik 1995) with a radial basis function (rbf) kernel. For a gene pair, the sum of the two corresponding embedding vectors was used as input. As in the cross-validation with GO, folds were split by gene (instead of by gene pair).

### 2.5 Cross-species gene set mapping

For each GO term in the slim set, annotated genes across the two species were considered as matched sets. We calculated a t-score for how closely annotated genes in one species were connected with annotated genes in the other species, while correcting for background network connectivity as in (Greene *et al.* 2015). The final z-score was calculated based on a comparison against a null distribution of gene sets matching in size and degree distribution to each GO term (sampled 100 times).

### 2.6 Clustering cross-species modules

The top 1% of all pairs in the score matrix were used to construct an unweighted and undirected graph between human and mouse, where edges are cross-species alignments between genes. We then applied Louvain (Blondel *et al.* 2008) to cluster the network vertices and visualized these clusters using Gephi (Bastian *et al.* 2009) with the OpenOrd (Martin *et al.* 2011) layout algorithm (cut parameter = 0.6). Vertices with degrees smaller than 20 were omitted from the final network. For each cluster, we calculated enrichment of GO terms (human and mouse), human OMIM (Hamosh *et al.* 2005) and GWAS (Manolio 2010) disease gene sets, and known human drug targets from DrugBank (Wishart *et al.* 2006) using the hypergeometric test. All resulting *P*-values were corrected for multiple hypothesis testing using Benjamini–Hochberg (Benjamini and Hochberg 1995).

### 2.7 Existing network alignment methods

We compared ETNA against three existing network alignment methods, MUNK (Fan *et al.* 2019), IsoRank (Singh *et al.* 2008), and HubAlign (Hashemifar and Xu 2014). We applied 5-fold cross-validation using the GO functional standard to choose hyperparameters for MUNK exactly as we did for ETNA. Both HubAlign and IsoRank had more run-time limitations. We were able to decrease our search space from 100 different hyperparameter settings to 10 to choose hyperparameters for HubAlign. For the 10 sets of different hyperparameters, we observed that HubAlign’s performance did not vary greatly so the reported performance should be reasonably optimized. However, because IsoRank needed several days to converge, default parameters were used.

We tried to compare our results with the other network alignment methods that do not use GO as input (for cross-species anchors, Table 1) but were unable to run PrimAlign (Kalecky and Cho 2018) (requires sequence alignment information for all gene pairs) and L-GRAAL (Malod-Dognin and Pržulj 2015) (error in the code). We were also unable to find a way to give sequence similarity as input to SANA’s (Mamano and Hayes 2017) code implementation and thus have also excluded it from the comparisons.

## 3 Results

### 3.1 ETNA outperforms existing network alignment and embedding methods in capturing functional similarity

We evaluated ETNA’s ability to capture functional similarity given only PPI data and sequence-based orthologous gene pairs. Since the majority of functional knowledge transfer tasks involve starting or ending with a human phenotype, we used ETNA to identify joint embeddings between human and four of the major model organisms: *M. musculus*, *S. cerevisiae*, *D. melanogaster*, and *C. elegans*. For evaluation, we took advantage of the fact that genes across species have been annotated to the same GO terms.

More specifically, to evaluate whether ETNA’s joint embedding captures functionally related gene pairs across species, we examined the predictive performance of the pairwise similarity score matrix (step 3 in Fig. 1) against a gold standard based on co-annotation of genes to the same GO term. We compared ETNA with the predictions made by MUNK (Fan *et al.* 2019), IsoRank (Singh *et al.* 2008), and HubAlign (Hashemifar and Xu 2014). MUNK’s predictions are directed from a source organism to a target organism, so we compared ETNA with both of MUNK’s directions.

ETNA generates a more functionally accurate embedding in all tested species pairs, especially for organisms with more complete PPI networks (Table 2, Supplementary Table S3, Supplementary Fig. S3). *S. cerevisiae* has the most complete PPI network among all model organisms and is where ETNA has the largest performance improvement over previous methods. Both *D. melanogaster* and *C. elegans* have very sparse PPI networks (density <0.2%), and their networks only cover a limited number of protein coding genes. Performance drops for all methods, and the difference between ETNA and other methods is also correspondingly smaller. Overall, these results suggest that with only PPI and sequence ortholog information, ETNA can infer the functional information between genes across species, and the predictive performance of ETNA improves quickly as PPI networks become more complete.

**Table 2.** AUPRC over random of ETNA, MUNK, IsoRank, and HubAlign for predicting cross-species gene pairs that share GO annotations based on 5-fold cross validation.^a^

Species pair	AUPRC (over random)			
	ETNA	MUNK	IsoRank	HubAlign
*H. sapiens* →*M. musculus*		0.590		
*H. sapiens* ←*M. musculus*	**0.805**	0.452	0.561	0.459
*H. sapiens* →*S. cerevisiae*		0.852		
*H. sapiens* ←*S. cerevisiae*	**1.390**	0.927	0.775	0.726
*H. sapiens* →*D. melanogaster*		0.607		
*H. sapiens* ←*D. melanogaster*	**0.724**	0.650	0.594	0.532
*H. sapiens* →*C. elegans*		0.515		
*H. sapiens* ←*C. elegans*	**0.572**	0.444	0.221	0.370

a AUPRC over random ${(\text{log}\;}_{2}(\frac{\text{AUPRC}}{\text{prior}}))$ is calculated to facilitate comparisons because each evaluation task has a different prior [proportion of positive examples in the gold standard (Section 2.3); the prior is different across organism pairs due to variability in the coverage of GO annotations within each organism, likely from differing levels of research and curation activity]. Thus, a score of 0 corresponds to random performance, and a score of 1 to a 2-fold improvement over random. For the four pairs of species (*H. sapiens*–*M. musculus*, *H. sapiens*–*S. cerevisiae*, *H. sapiens*–*D. melanogaster*, *H. sapiens*–*C. elegans*), the random priors are 0.059, 0.043, 0.044, and 0.044 respectively. Because MUNK’s predictions require choosing a source organism and a target organism, we present its performance for both directions (the arrow points from source to target). For each species pair, the top performance is shown in bold.

A gene can have multiple functions; thus, the functional similarity between a pair of genes is more complex than the presence or absence of a single shared GO annotation. To capture this, we also used the Jaccard index to quantify how much functionality (multiple GO terms) is preserved for cross-species gene pairs. Intuitively, two genes that share the same profile of GO terms will have a Jaccard index of 1. On the other hand, if two genes are both annotated to several distinct GO terms but happen to share one annotation, using the Jaccard index will downweight their similarity by the total number of annotations. In addition to MUNK, IsoRank, and HubAlign, we also generated two additional baselines: gene pairs ranked by degree and random gene pairs. All of the methods, including ranking by degree, have a higher Jaccard index for higher ranked pairs and gradually converge to random at around 5% of all pairs (Supplementary Fig. S5).

**Figure 2.**
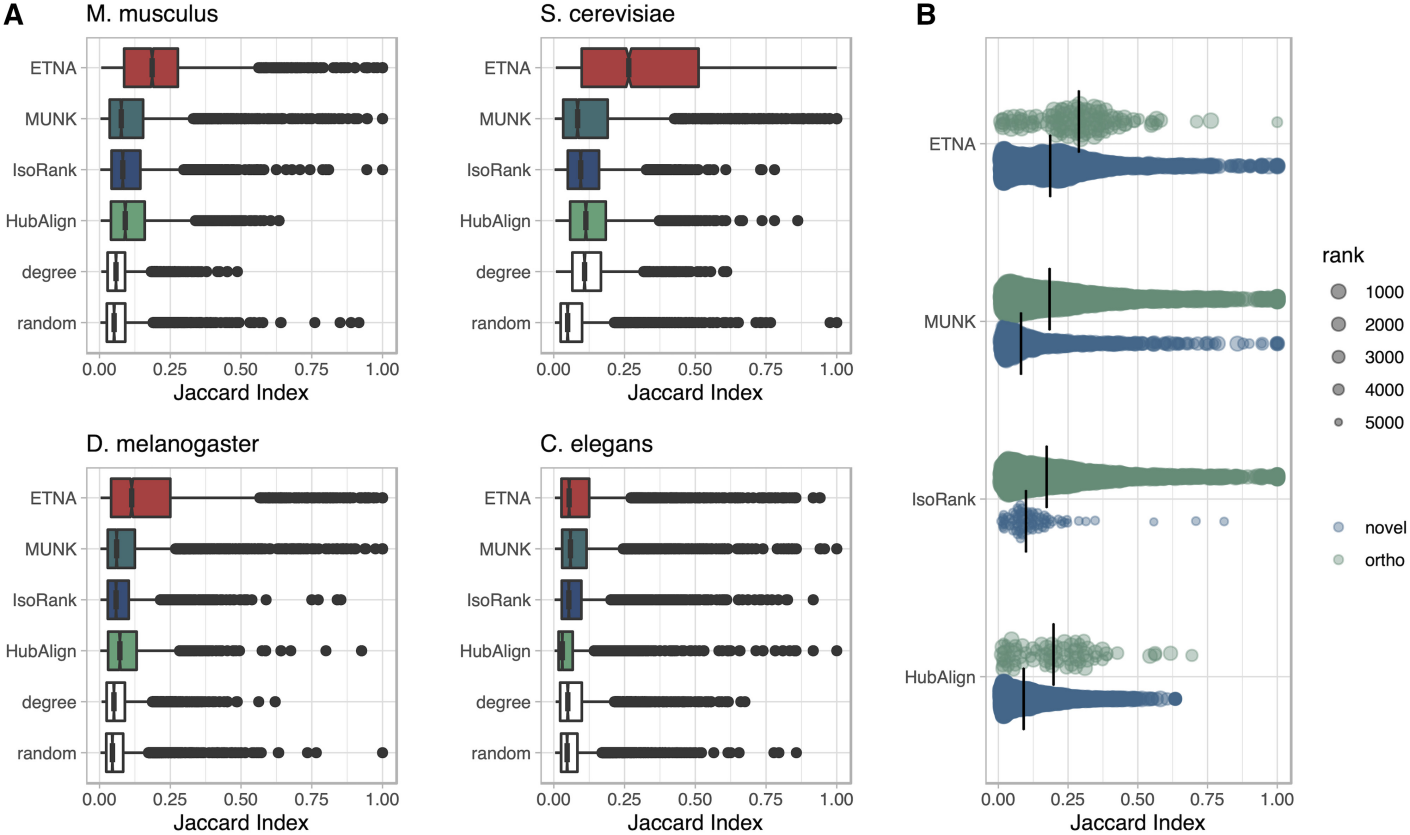
Jaccard index for top 5000 aligned pairs. (A) Box plots for top 5000 gene pairs for *H.sapiens* and four model organisms (*M. musculus*, *S. cerevisiae*, *D. melanogaster*, *C. elegans*). Four methods (ETNA, MUNK, HubAlign, IsoRank) and two baselines (degree and random) are compared. ETNA has the best performance in *M. musculus* (*P* < 10^–16^), *S. cerevisiae* (*P* < 10^–16^), *D. melanogaster* (*P* < 10^–16^), and comparable performance to MUNK in *C.elegans*, demonstrating how ETNA’s joint embedding captures multi-functional similarity. All *P*-values reported are from Wilcoxon rank-sum test (Conover 1999) to the second best method. (B) Jaccard index comparison between orthologous (ortho) pairs (in green, top) and nonorthologous (novel) pairs (in blue, bottom) within top 5000 pairs of *H. sapiens*–*M. musculus* alignment. ETNA is better at prioritizing gene pairs beyond orthologs.

In examining the Jaccard index between the top 5000 ranked pairs of genes (without orthologs used for alignment) from different methods, we found that ETNA consistently outperforms other methods across species pairs (Fig. 2A). Note that this trend is also consistent across top ranked pairs (Supplementary Fig. S4), with and without orthologs, though we only show 5000 of them (top ranked pairs) here. For the sparsest PPI network, *C. elegans*, ETNA has comparable performance to MUNK and is significantly better than the other network alignment methods and baselines (*P* < 10^–6^). ETNA significantly outperforms all previous methods (one-sided Wilcoxon rank-sum *P* < 10^–16^) in predicting multifunctional similarity between *H. sapiens* with *M. musculus*, *S. cerevisiae*, and *D. melanogaster*. This demonstrates that ETNA’s joint embedding cannot only reflect whether two genes are related, but also quantify the extent of their relationship.

When conducting this evaluation, we observed that two of the methods, IsoRank and MUNK, strongly prioritized the orthologs used to anchor the alignment across a species pair (Fig. 2B). Unlike other methods, ETNA uses pairwise orthology information alone rather than sequence similarity across all pairs of genes. As IsoRank linearly combines sequence information with topological structures in its algorithm, orthologous pairs can be particularly favored in the alignment. On the other hand, because nonorthologous pairs (most of the pairs) naturally have less sequence similarity information, they are mostly not prioritized. To systematically explore these trends, we also compared each method’s performance with and without orthologous pairs (Fig. 2B, *H. sapiens*–*M. musculus* alignment; Supplementary Fig. S6, all other alignments), discovering that ETNA has consistently good performance, beyond prioritizing orthologous pairs alone.

### 3.2 ETNA enables cross-species prediction of genetic interactions

One advantage of a cross-species joint embedding is that we can detect relationships beyond functional similarity captured by GO. Synthetic lethality (SL) is a type of genetic interaction, when two (or more) perturbing genes cause a deleterious effect on the organism that is unseen in the respective single gene perturbations. SL has received increasing attention for its potential application to cancer treatments (O’Neil *et al.* 2017), but the experimental detection of SL can be costly and time consuming to perform, especially on higher order organisms.

The systematic probing of genetic interactions in *S. cerevisiae* (*Sce*) (Tong *et al.* 2004, Costanzo *et al.* 2010) have yielded rich datasets of SL relationships. Meanwhile, similar, smaller scale efforts in a distantly related yeast, *S. pombe* (*Spo*), have resulted in interesting network comparisons (Dixon *et al.* 2008), which together, provide a valuable resource to evaluate the cross-species predictive power of ETNA. We were also curious to what extent SL relationships in *Sce* could be used to predict the more limited number of *H. sapiens* (*Hsa*) experimental SL data (Kessler *et al.* 2012, Bailey *et al.* 2015, De Kegel *et al.* 2021).

To this end, we explored two paradigms for using cross-species embeddings to predict SL:

1. Using SL pairs from both organisms to predict a held out set of SL relationships
2. Using SL pairs reported in one species to predict the held out SL relationships in the other

This second paradigm is clearly more challenging, but also more closely mimics how we may use these embeddings in practice, where specific types of data may only be richly available in one model organism. We evaluate how well we can “transfer” the wealth of genetic interactions captured in one species to the other species using only PPI and sequence information, which are more readily available across species. ETNA shows a significant performance increase across the board in comparison to the only other existing method that provides a joint embedding that can be used for downstream tasks, MUNK (regardless of which directionality of MUNK was used) (Table 3).

**Table 3.** ETNA consistently outperforms MUNK in prediction of genetic interactions between *S. cerevisiae* (*Sce*) ↔*S. pombe* and (*Spo*) and *S. cerevisiae* (*Sce*) ↔*H. sapiens* (*Hsa*).^a^

SL prediction task	AUPRC			AUROC		
	ETNA	MUNK		ETNA	MUNK	
	*Sce* ↔*Spo*	*Sce* →*Spo*	*Sce* ←*Spo*	*Sce* ↔*Spo*	*Sce* →*Spo*	*Sce* ←*Spo*
*Sce* + *Spo* →*Sce*	**0.757**	0.681	0.655	**0.753**	0.700	0.650
*Sce* + *Spo* →*Spo*	**0.773**	0.606	0.554	**0.744**	0.581	0.571
*Sce* →*Spo*	**0.762**	-	-	**0.741**	-	-
*Spo* →*Sce*	**0.688**	0.533	0.543	**0.648**	0.499	0.431
	*Sce* ↔*Hsa*	*Sce* →*Hsa*	*Sce* ←*Hsa*	*Sce* ↔*Hsa*	*Sce* →*Hsa*	*Sce* ←*Hsa*
*Sce* + *Hsa* →*Sce*	**0.781**	0.675	0.652	**0.774**	0.683	0.677
*Sce* + *Hsa* →*Hsa*	**0.738**	0.485	0.553	**0.803**	0.396	0.582
*Sce* →*Hsa*	**0.650**	-	-	**0.687**	-	-
*Hsa* →*Sce*	**0.686**	0.552	0.642	**0.684**	0.545	0.664

a For each SL prediction task, A→B indicates SL pairs in *A* were used for training to predict SL pairs in *B* (e.g. *Sce* + *Spo* →*Sce* indicates that SL gene pairs from both *Sce* and *Spo* were used for training, and a set of held out SL gene pairs in *Sce* were used for evaluation). When SL pairs for two organisms are used for training, equal numbers of positives were used (i.e. SL pairs were subsampled for the larger of the two organisms). Notably, ETNA is able to predict genetic interactions across species well using *only* interactions reported in the other (*Sce* →*Spo*, *Spo* →*Sce*, *Hsa* →*Sce*, and *Sce* →*Hsa*). Each of the gold standards has balanced positives and negatives (prior = 0.5). For prediction tasks based on *Sce* examples, MUNK failed to converge after several days of training so we were unable to calculate prediction performance.

As expected, the predictive performance for predicting one species’ SL relationships entirely based on examples from the other species is lower than using SL information from both species as input. Nevertheless, ETNA still performs very well and also significantly better than MUNK. Due to MUNK’s large embedding size, as well as the large number of SL pairs reported in *Sce* (13,920 gene pairs), MUNK failed to converge even after several days. To enable comparisons of ETNA with MUNK for training on *Sce*, we also subsampled the number of *Sce* SL pairs used for training to be the same number as the target organism (*Spo*: 1,078 gene pairs, *Hsa*: 1,883 gene pairs, Supplementary Table S5). By subsampling the training set, we were able to obtain predictions from MUNK. We see that in this direct comparison, ETNA still consistently outperforms MUNK, though, as expected, there is a performance drop compared with using the full set of available *Sce* training examples (Table 3). In addition to demonstrating the strong predictive performance of ETNA’s embeddings, this analysis emphasizes how being able to customize embedding sizes in ETNA is critical for downstream analyses, as it enables computationally tractable prediction tasks that can leverage the full set of training examples.

Notably, we also noticed MUNK’s joint embeddings can result in inconsistent predictions between the two alignment directions, and furthermore, sometimes the nonintuitive direction may perform better for a particular task. For example, after subsampling the gold standard for computational tractability, when attempting to predict *Hsa* using only *Sce* gene pairs (*Sce* →*Hsa*), MUNK’s *Sce* ←*Hsa* embedding performs better, whereas the embedding that matches the direction of the prediction task has poor performance (Supplementary Table S5). This highlights the importance of having a bidirectional embedding. Together, these results demonstrate how ETNA enables the knowledge transfer of genetic interactions from a well-studied species to another.

### 3.3 Cross-species alignment improves predictive performance of individual embeddings

To identify a joint network embedding, ETNA iterates between calculating individual embeddings (step 1) and performing cross-training (step 2) to align the two spaces (Fig. 1). This gives ETNA the opportunity to use information from the other species to refine the encoding of individual networks. We performed an ablation study on the individual network embeddings to dissect the contribution of the cross-training process and the NetMF matrix input *M*. To this end, we explored the extent to which the individual network embeddings captured functional signal in the original species (as opposed to the species that it is being aligned to), with and without cross-training. We also checked the performance of still including cross-training, but directly using the adjacency matrix as input to the autoencoders instead of the NetMF matrix *M* (Equation 3 in Section 2). In each paradigm, we calculated the cosine similarity of latent embeddings for all gene pairs within one species. We evaluated these similarity scores for their ability to recapitulate shared Gene Ontology (GO) annotations between genes within the respective species.

**Figure 3.**
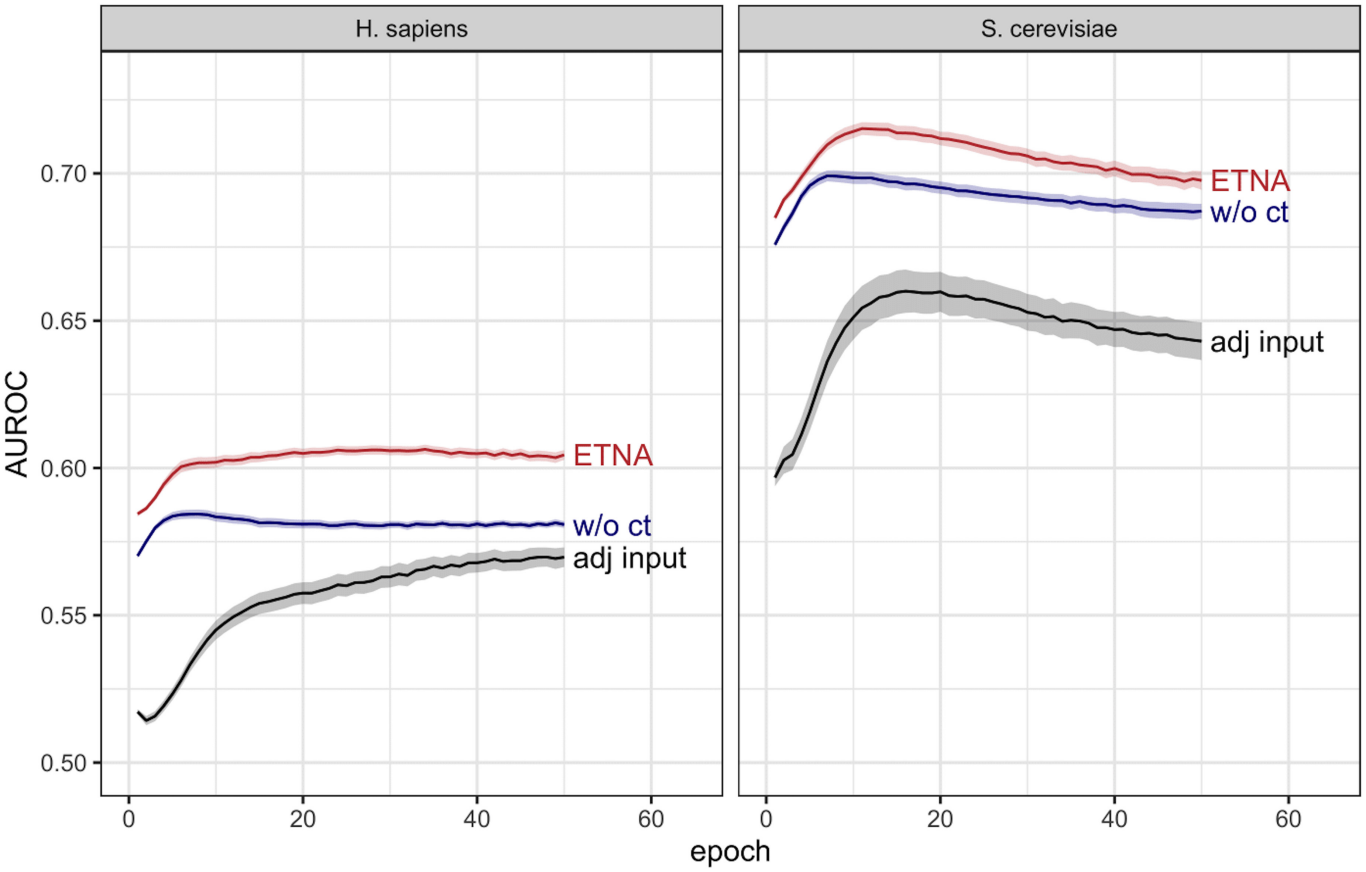
Predicting functional similarity using individual network embeddings in *H. sapiens* and *S. cerevisiae* with ETNA (red), embeddings without cross-training (blue, ‘w/o ct’), and using the adjacency matrix as input (black, ‘adj input’). Lines show mean AUROC across 100 random sets of hyperparameters, and ribbons denote the 95% confidence interval for predicting functional similarity [defined based on co-annotation to the same GO term for all terms in the gold standard (Section 2.3)]. Cross-training and using the NetMF matrix instead of the adjacency matrix as input both lead to significant improvements to ETNA’s predictive performance.

Interestingly, we discovered that the alignment process not only preserves the existing functional relationship captured by the individual network embeddings, but also further improves the predictive performance of the individual embeddings using information from the other network (Fig. 3). This suggests that while finding an alignment between two spaces, ETNA’s cross-training step also enables each individual network to leverage the information in the other network to refine its own embeddings. This intuition may also be why cross-training yields a larger performance improvement for *H. sapiens* than for *S. cerevisiae*, since *S. cerevisiae* has a much more complete PPI network. We observe a similar trend when comparing ETNA with a modified version that uses the adjacency matrix (instead of the NetMF matrix) as input. The NetMF matrix is able to capture more distant relationships between genes, beyond simply that of the direct neighbors, and thus improves the performance of the network embeddings dramatically.

### 3.4 ETNA’s joint embedding space captures functional similarity of gene sets

Researchers also often encounter gene sets of interest, and as many important biological processes are performed by the cooperation of multiple proteins, beyond matching gene pairs between two species, it is important to explore the functional alignment of gene sets. We thus explored whether genes annotated to the same GO term across two species were more significantly connected to each other than expected by random.

**Figure 4.**
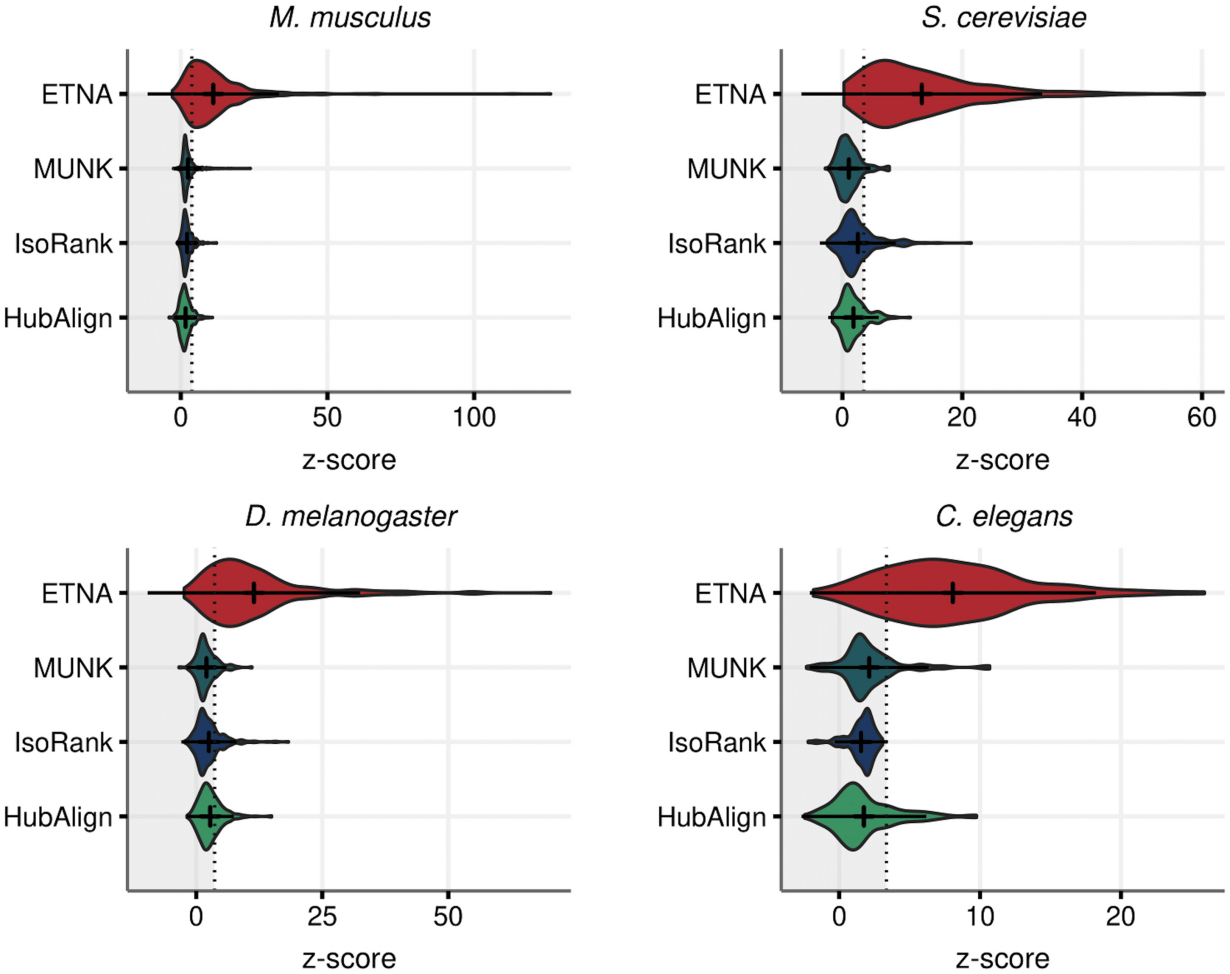
Evaluating cross-species GO term matching demonstrates that functional gene sets have consistently stronger correspondence than other methods. The alignment between *H. sapiens* and four model organisms (*M. musculus*, *S. cerevisiae*, *D. melanogaster*, *C. elegans*) are considered. There are 645, 292, 349, and 129 shared GO terms, respectively. We calculated a *z*-score for connectivity between genes annotated to the same GO term across species against a null distribution generated from random degree-matched gene sets of the same size. The dotted line is the *z*-score that corresponds to the Bonferroni corrected *P*-value=.05.

To this end, as in Greene *et al.* (2015), we compared the connectivity of the GO term split across species to a null distribution of random degree-matched gene sets of the same size. Across the board, ETNA was able to identify significant [after Bonferroni correction (Dunn 1961, Korthauer *et al.* 2019)] matches for over 75% of the GO terms shared between species (*H. sapiens*–*S. cerevisiae*: 94%, *H. sapiens*–*M. musculus*: 75%, *H. sapiens*–*D. melanogaster*: 77%, and *H. sapiens*–*C. elegans*: 82%), whereas other methods were only able to match ∼25% of the GO terms at the same significance threshold (Fig. 4). In this evaluation, we show that ETNA not only captures pairwise gene relationships between two species but also that functional groups are also meaningfully clustered in the joint embedding space.

By taking a closer look at these *z*-scores, we found that most of the high scoring GO terms described functions such as RNA polymerase, transcription, etc. These functions have been shown to be highly conserved through evolution across eukaryotic organisms. As for GO terms that had a z-score below the Bonferroni threshold, many were child nodes under the umbrella GO term “response to stimulus.” One hypothesis for why ETNA does not capture these functions as well is that stimulus response may achieved through other types of gene/protein interactions (e.g. via signal transduction, phosphorylation) rather than physically interacting, so when only considering the PPI network as input, ETNA could miss these relationships.

### 3.5 ETNA can reveal shared mechanisms of fundamental biological processes and disease pathology

**Figure 5.**
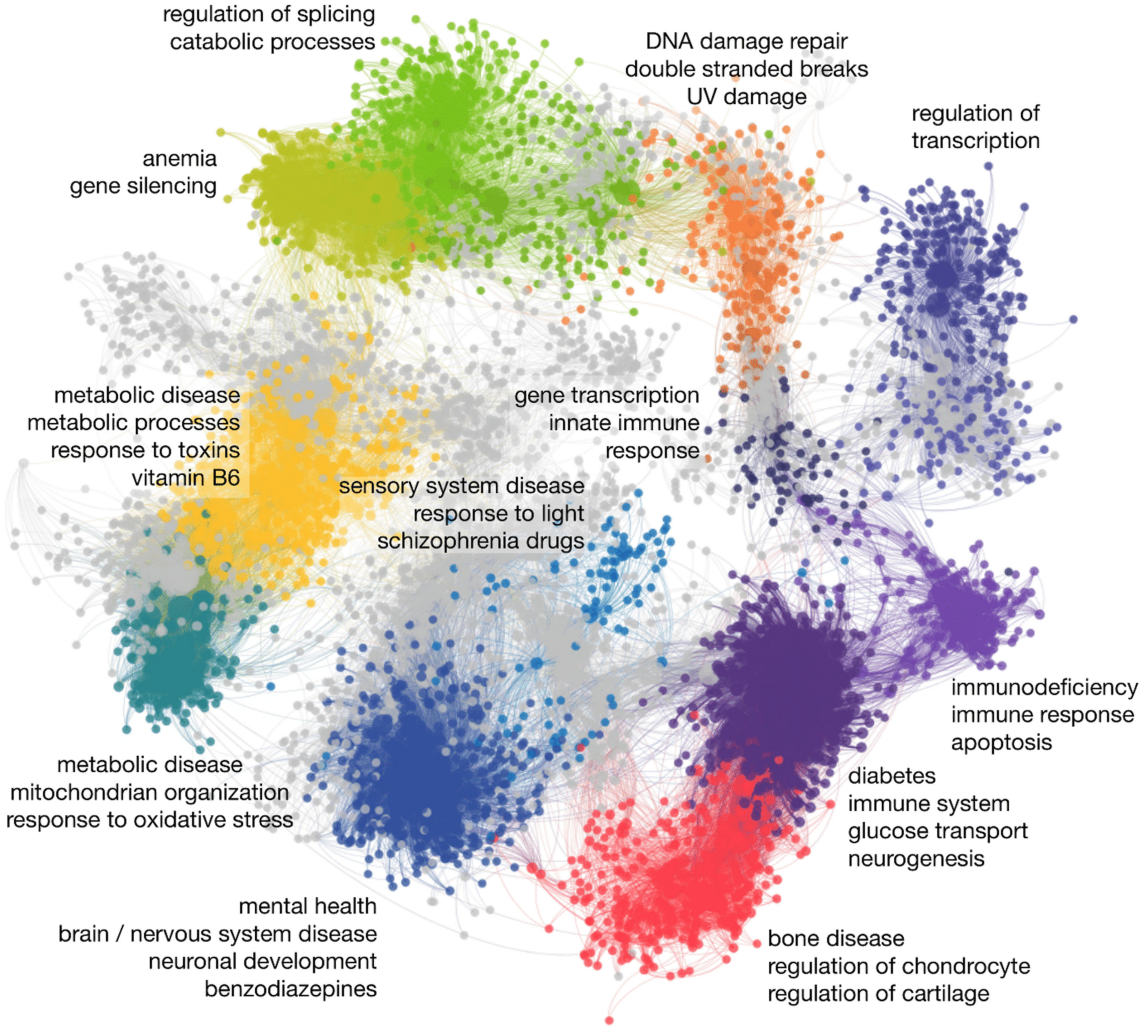
Network of the top gene mappings between human and mouse. This network shows the top 1% of ETNA scores for the alignment of *H. sapiens* and *M. musculus*, where each vertex is a gene and each edge is an alignment between the two species. Enrichment of disease, drug, and GO terms were calculated for each cluster, and selected enriched clusters are colored and labeled here with significant terms summarized, highlighting the potential for ETNA to discover novel biology. Clusters with similar colors have shared functions.

So far, our evaluations have shown that ETNA captures known functional biological processes. Taking a closer look at the alignment between human and mouse, we wanted to explore whether the top gene pairings can be used for functional knowledge transfer of other gene sets. Thus, we took the top 1% of ETNA scores between human and mouse and clustered the data, identifying functional modules. We found 40 modules with at least 20 edges and performed enrichment on GO terms (human and mouse), drug targets [DrugBank (Wishart *et al.* 2006)], and known human disease genes [annotated in OMIM (Hamosh *et al.* 2005) and GWAS (Manolio 2010)]. There were a range of significantly enriched terms in most modules, but here we highlight some of the top clusters with interesting functions as a proof of concept with the full set (Supplementary data). These modules covered a range of disease mechanisms and key conserved core biological processes, including transcription regulation, splicing, and DNA damage repair (Fig. 5).

The network modules are roughly co-located with others of similar function. For example, the two purple clusters are enriched for immune-related GO terms, with the bottom module also enriched for glucose-related terms and diabetes, and the upper module enriched for diseases related to immunodeficiency. The darker purple module under the orange module is also enriched for a distinct set of immune processes as well as transcription-related GO terms and is located somewhat between the immune modules and the regulation-specific module. Another interesting cluster (blue, bottom-left) is enriched for neuronal development related GO terms, mental health diseases, and benzodiazepines, a class of drugs used to treat anxiety. Finally, the blue cluster in the middle, while near the large neuronal development cluster, is a distinct region of enrichment for neurological processes pertaining to light processing and sensory system disease, as well as drugs used to treat schizophrenia. Schizophrenia patients often have sensory overload and their failure to handle environmental stimuli is one of the key facets of the disease (Javitt 2009). This also suggests that ETNA may uncover shared mechanisms between diseases and drugs in new ways given additional information from other species. While we only highlight a few specific examples here, there are many more interesting connections that are ripe for exploration, and as such, all models, embeddings, scores, and code are available for download at https://github.com/ylaboratory/ETNA.

## 4 Discussion

In this study, we introduce ETNA as a method to transfer functional information across species. Unlike traditional network alignment methods that calculate a single score to capture similarity between a pair of genes, ETNA generates a general purpose joint embedding, capturing functional relevance between two genes as multi-dimensional vectors. Instead of linearly combining topological structure and orthologous information, ETNA introduces an autoencoder-based framework that captures the nonlinearities, as well as local and global relationships in network topology, then uses cross-training to construct a joint embedding.

We have demonstrated that ETNA is capable of capturing both pairwise and group functional relationships between human and other model organisms. Beyond inferring unannotated gene functions from their closely related genes in other organisms, ETNA’s embedding enables transfer of genetic interaction knowledge from one species to another. As the number of possible pairs of genetic interactions has a combinatorial relationship with the number of genes, and gene knockout can be costly or intractable to perform at scale on higher-order organisms, ETNA’s joint embedding provides a new way to unravel gene relationships that are difficult to detect experimentally. Finally, by exploring the human-mouse functional landscape, we are able to identify interesting connections between mouse functional studies with complex human diseases, setting the stage for potential opportunities for translational studies.

Though we have applied ETNA to PPI networks here, the methodological framework can be easily applied to other types of biological networks. As shown in Table 2, the more complete a PPI network is, the better ETNA can use this information to create a more accurate joint embedding, so it would be interesting to explore whether using predicted PPIs to supplement experimentally derived networks would improve performance. But beyond PPI networks, we can envision alignment of metabolic or regulatory networks. Integrated functional networks (Wong *et al.* 2015) designed to predict functional similarities would also be natural to use as input into ETNA. In addition, currently ETNA uses orthology information as “anchors” to guide the cross-training and alignment between networks. An interesting extension of ETNA is to use weighted sequence similarities (or even other types of anchor similarities) to guide the cross-training step.

Furthermore, because the joint embedding of ETNA does not require choosing a source and target, it opens possibilities for extending the framework to simultaneously perform alignment for more than two species. We have found here that the cross-training step enables ETNA to use information from other species to refine individual embeddings, so we anticipate that a “multiple-species network alignment” could result in an even more accurate joint embedding and enable placing model systems with limited experimental studies into the functional landscape.

## Supplementary Material

btad529_Supplementary_Data

## Data Availability

No new data were generated in support of this research.
