# Supplementary material for: Joint embedding of biological networks for cross-species functional alignment
Source: Bioinformatics. 2023 Aug 26;39(9):btad529. doi: 10.1093/bioinformatics/btad529 (PMC10477935; doi:10.1093/bioinformatics/btad529)
Supplement: btad529_Supplementary_Data [file btad529_supplementary_data.pdf]

Additional Files

Table S1. Selected hyperparameters for ETNA models between *H. sapiens* (*Hsa*) and four model organisms: *M. musculus* (*Mmu*), *S. cerevisiae* (*Sce*), *D. melanogaster* (*Dme*), and *C. elegans* (*Cel*). α, γ, ω, and λ are described in Equation (1) and ϕ is described in Equation (5). These parameters were searched on a logarithmic scale (base 10). Each individual species embedding has its own α, γ, ω, and λ parameters. For each joint embedding listed in the table, the first column corresponds to the hyperparameters for the *H. sapiens* embedding, and the second corresponds to that of the model organism.

	search space	*Hsa* ↔ *Mmu*		*Hsa* ↔ *Sce*		*Hsa* ↔ *Dme*		*Hsa* ↔ *Cel*	
α	$10^x; x \in [0,3]$	0.79	2.86	1.22	2.62	2.48	2.60	2.29	1.48
γ	$10^x; x \in [-1,2]$	-0.31	0.77	0.49	-0.42	-0.07	-0.31	1.05	0.11
ω	$10^x; x \in [-1,2]$	0.73	0.4	1.03	-0.23	-0.89	1.39	0.93	-0.24
λ	$10^x; x \in [-1,2]$	0.05	-0.91	-0.92	-0.45	0.84	0.99	0.60	-0.75
ϕ	$10^x; x \in [-1,2]$	1.72		1.55		0.31		2.92	
epoch	$[5,30]$	5		8		15		19	

Table S2. Summary statistics of PPI networks and GO annotation information. For each PPI network, the # of vertices and # edges as well as corresponding density (i.e., % of edges out of possible number of edges based on # of vertices) is provided. Furthermore, for each organism, we estimate the % of genome covered by at least one measured PPI relationship (i.e., (# vertices) / (# estimated protein-coding genes) for each species), the # of orthologs with *H. sapiens*, and the % of genes that in the PPI network with at least 1 GO annotation.

	# vertices	# edges	density	% genome with a reported PPI	# orthologs with *H. sapiens*	% genes with a GO annotation
H. sapiens	17,120	418,512	0.286%	81.5%	NA	51.1%
M. musculus	7,763	47,833	0.159%	38.8%	10,820	60.6%
S. cerevisiae	5,669	110,776	0.689%	85.9%	2,221	77.6%
D. melanogaster	7,711	49,769	0.167%	55.1%	4,603	64.7%
C. elegans	4,439	18,301	0.186%	22.2%	2,561	63.1%

Table S3. AUROC of ETNA, MUNK, IsoRank, and HubAlign for predicting cross-species gene pairs that share GO annotations based on 5-fold cross validation. Because MUNK's predictions require choosing a source organism and a target organism, we present its performance for both directions (the arrow points from source to target).

Species Pair	AUROC			
	ETNA	MUNK	IsoRank	HubAlign
H. sapiens → *M. musculus*<br>*H. sapiens* ← *M. musculus*	**0.604**	0.589<br>0.557	0.589	0.566
H. sapiens → *S. cerevisiae*<br>*H. sapiens* ← *S. cerevisiae*	**0.665**	0.626<br>0.629	0.620	0.610
H. sapiens → *D. melanogaster*<br>*H. sapiens* ← *D. melanogaster*	**0.601**	0.597<br>0.607	0.594	0.585
H. sapiens → *C. elegans*<br>*H. sapiens* ← *C. elegans*	0.593	0.572<br>**0.596**	0.563	0.536

Table S4. Mean validation error and mean test error of ETNA on 4 species pairs based on 5-fold cross validation. AUPRC over random calculated as in Table 2.

Species Pair	AUPRC (over random)		AUROC	
	validation	test	validation	test
H. sapiens - M. musculus	0.809	0.805	0.605	0.604
H. sapiens - S. cerevisiae	1.395	1.390	0.665	0.665
H. sapiens - D. melanogaster	0.726	0.724	0.601	0.601
H. sapiens - C. elegans	0.583	0.572	0.594	0.593

Table S5. Prediction of genetic interactions from *S. cerevisiae* (*Sce*) to *S. pombe* (*Spo*) and *H. sapiens* (*Hsa*), where the *Sce* training set is subsampled to the size of the target organism. For each SL prediction task, $A \rightarrow B$ indicates SL pairs in A were used for training to predict SL pairs in B. Here, instead of using the entirety of the original 13,920 training examples in *Sce*, the training set as subsampled to match the training set size for *Spo* and *Hsa* (1,078 and 1,883 examples, respecitvely).

SL Prediction Task	AUPRC ETNA	AUPRC MUNK		AUROC ETNA	AUROC MUNK	
	Sce ↔ *Spo*	*Sce* → *Spo*	*Sce* ← *Spo*	*Sce* ↔ *Spo*	*Sce* → *Spo*	*Sce* ← *Spo*
Sce → *Spo*	**0.713**	0.586	0.567	**0.715**	0.582	0.564
	Sce ↔ *Hsa*	*Sce* → *Hsa*	*Sce* ← *Hsa*	*Sce* ↔ *Hsa*	*Sce* → *Hsa*	*Sce* ← *Hsa*
Sce → *Hsa*	**0.594**	0.449	0.548	**0.608**	0.376	0.577

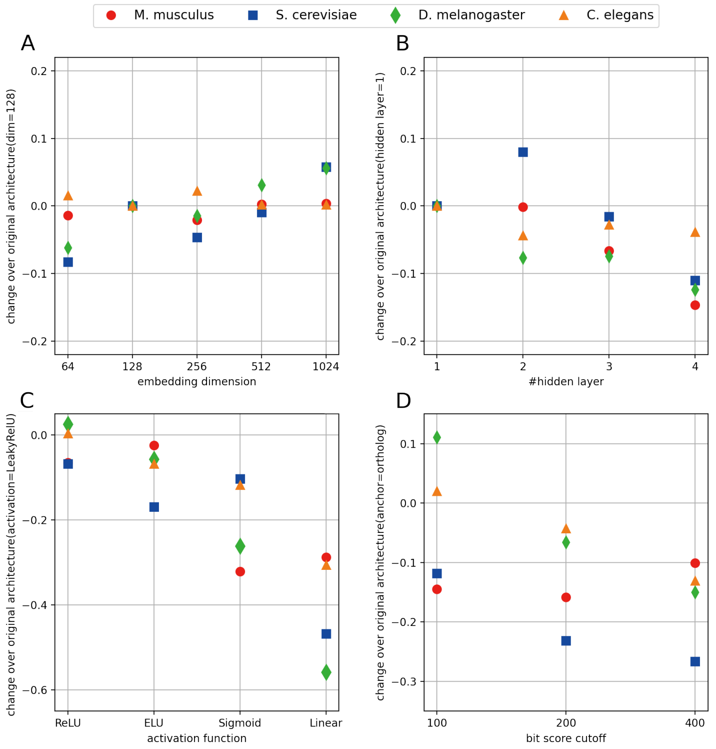

Figure S1. ETNA's performance is robust to different neural network architecture and choice of anchors. In each subfigure, the change in prediction performance using the alternative neural network architecture is shown. The change shown is the $\log_2$ fold change over the original AUPRC. **(A)** Performance change with respect to different embedding dimensions (64, 128, 256, 512, 1024), where ETNA's default embedding dimension is 128. **(B)** Performance change with respect to different numbers of hidden layers (1, 2, 3, 4), where ETNA's original architecture had 1 hidden layer. **(C)** Performance change with respect to different choices of activation function used (ReLU, ELU, Sigmoid, Linear), where ETNA's default is LeakyReLU with a negative slope of 0.1. **(D)** Performance change with respect to different choice of cross-species anchors, based on BLAST bit score cutoffs (100, 200, 400), instead of orthologs as is the default in ETNA.

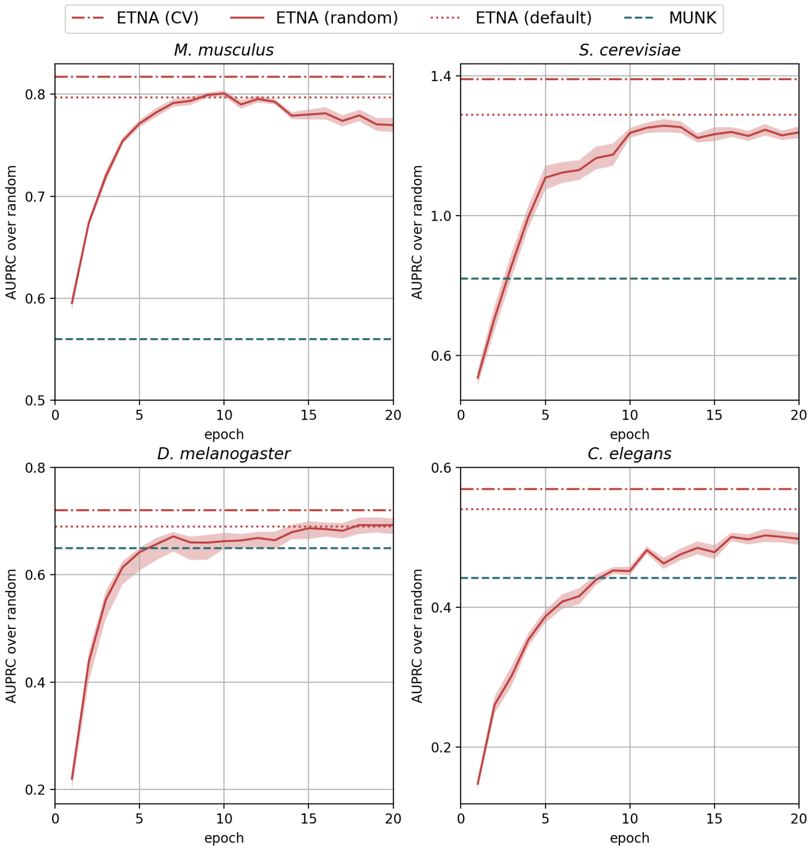

Figure S2. ETNA's performance is robust to choice of hyperparameters. The red solid line shows the mean AUPRC over random ($\log_2(\frac{\text{AUPRC}}{\text{prior}})$) based on 10 random sets of hyperparameters, and ribbons denote the 95% confidence interval. While hyperparameters selected via cross-validation (ETNA (CV)) performed the best, models with random hyperparameters (ETNA (random)) can also achieve strong performance, and within a few epochs of training, consistently outperform MUNK (the best performing previously existing method). Using ETNA with suggested default parameters (ETNA (default)) typically results in comparable to better performance than with random hyperparameters, and the performance gap with cross-validated hyperparameters is not large.

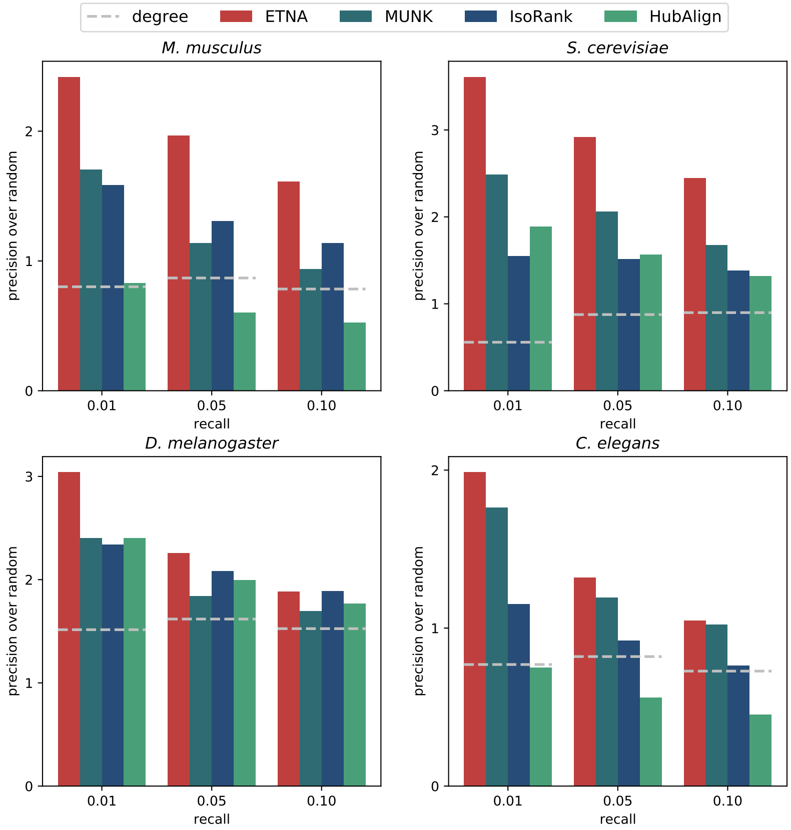

Figure S3. Precision over random at recall=0.01, 0.05, 0.10 of ETNA, MUNK, IsoRank, and HubAlign for predicting cross-species gene pairs that share GO annotations. As in Table 2, precision over random ($\log_2(\frac{\text{precision}}{\text{prior}})$) is calculated to facilitate comparisons because each evaluation task has a different prior. Degree represents the baseline metric of ranking gene pairs by simply by their degree in the PPI network.

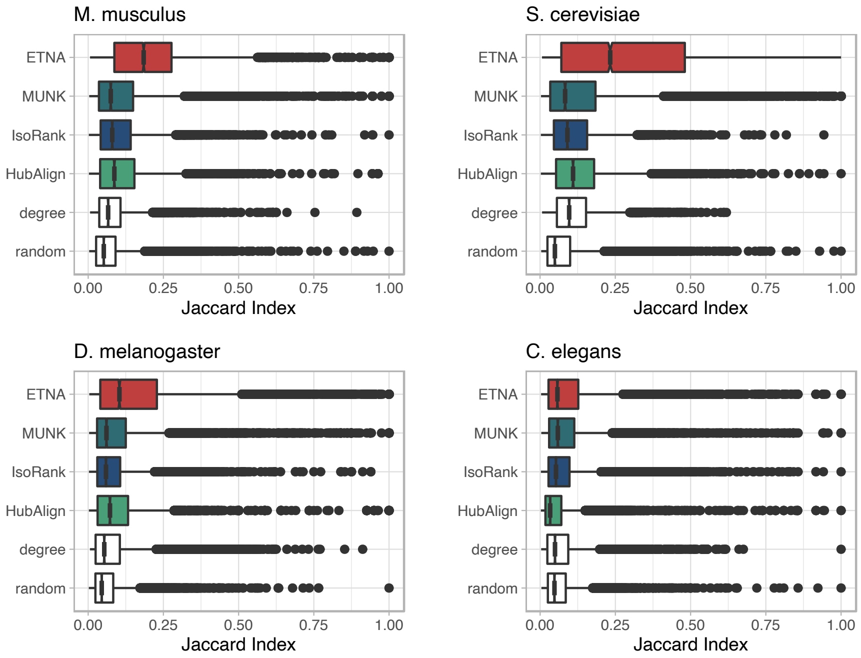

Figure S4. Jaccard index for top 10,000 aligned pairs.

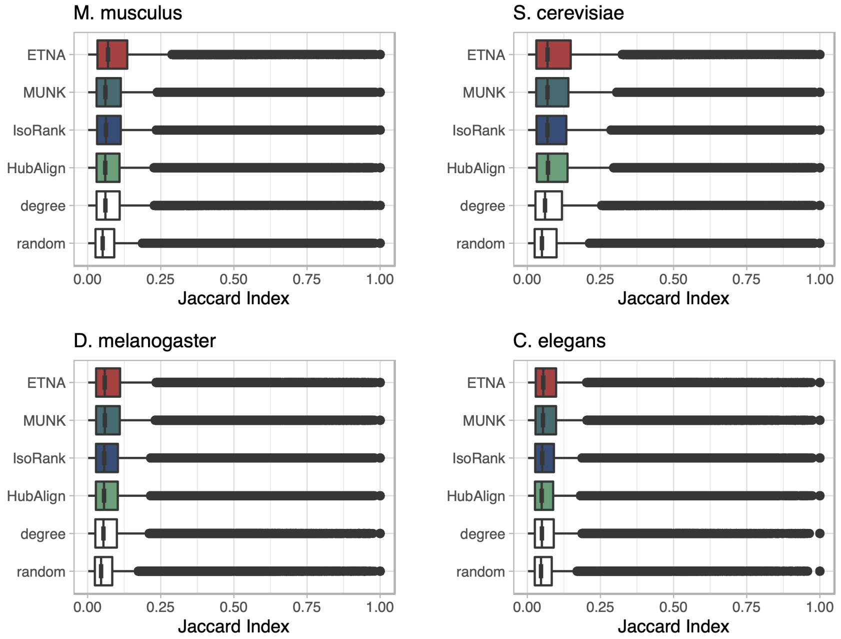

Figure S5. Jaccard index for top 5% aligned pairs.

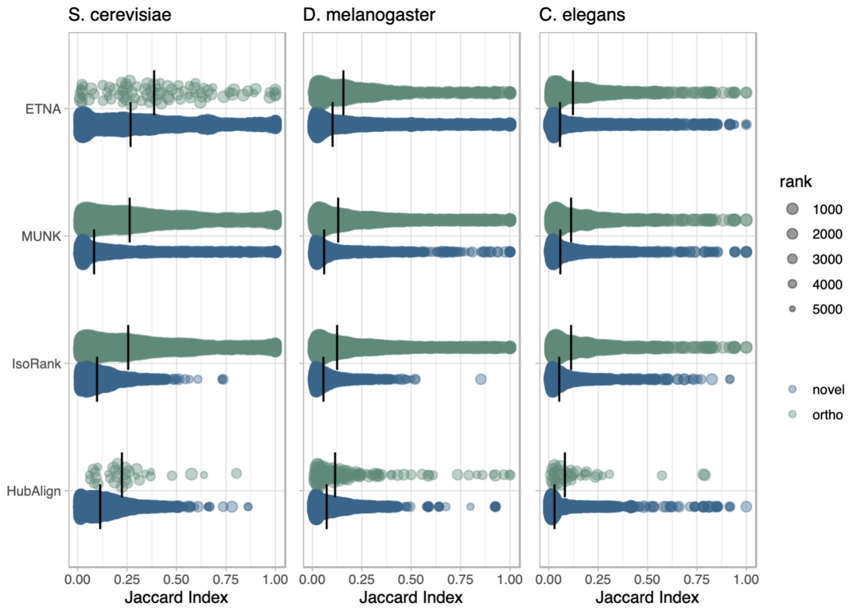

Figure S6. Jaccard index comparison between orthologous pairs and non-orthologous pairs within top 5,000 pairs.
